# Spectrum of acute hepatitis and its clinical outcome in a central region in Tunisia

**DOI:** 10.11604/pamj.2021.40.53.25725

**Published:** 2021-09-21

**Authors:** Nour Elleuch, Manel Moalla, Sana Mahmoud, Aya Hammami, Hanen Jaziri, Wafa Ben Ameur, Wafa Dahmani, Aida Ben Slama, Ahlem Brahem, Salem Ajmi, Mehdi Ksiaa, Ali Jmaa

**Affiliations:** 1 Department of Gastroenterology, Sahloul Hospital, Sousse, Tunisia

**Keywords:** Acute hepatitis, etiology, epidemiology, prognosis, Sousse

## Abstract

**Introduction:**

given the lack of studies on acute hepatitis (AH) in Tunisia, we carried out this study to find the etiological spectrum and clinical profile of AH and to investigate the impact of viral etiology on the outcomes of AH.

**Methods:**

retrospective descriptive study collecting all patients with AH from 2010 to 2017. The data were compared between two groups (viral AH and non-viral AH).

**Results:**

one hundred and three patient´s files were included. The average age of our patients was 30.15 years. An etiology was found in 92 patients (89.3%). The viral etiology was found in 70 patients (76.1%). Hepatitis A virus (HAV), hepatitis B virus (HBV), hepatitis C virus (HCV) and cytomegalovirus (CMV) were in the cause in 52, 16, 1 and 1 patient respectively. Elsewhere, it was toxic hepatitis in 10 patients (10.9%) including 7 of drug-related AH. Budd-Chiari syndrome and autoimmune hepatitis with acute onset were reported in 3 (3.3%) and 7 (7.6%) patients, respectively. Patients with viral AH were younger than those with non-viral AH (p = 10-^3^). There was more recourse to hospitalization for non-viral AH. Patients with viral AH had a higher mean aminotransferase (ALT) level than those with non-viral AH. The liver damage was more severe in the non-viral AH group with lower PT. There was more severe form, more transition to chronicity and more deaths in the non-viral AH group. **Conclusion:** the results found in our study concerning the distribution of the etiologies of AH as well as their evolutionary aspects are consistent with the data in the literature.

## Introduction

Acute hepatitis (AH) is an acute inflammation of the liver that can be complicated by an acute liver failure (ALF) characterized by an alteration of the synthetic functions of the liver, in particular coagulation factors and a decline in the capacity of degradation of ammonia leading to hepatic encephalopathy [1]. AH is a major public health problem, giving the risk of progression to severe hepatitis, chronic hepatitis or even cirrhosis. There are very few studies that have looked at the epidemiological profile of AH worldwide. The majority of studies were focused on ALF. The most commonly reported etiologies of AH are viral and drug-related ones, but in 15% of cases the cause remains unknown [2]. In Tunisia, there are only very few studies which looked at the etiological profile of AH and which are quite old, with a small population study [3]. An epidemiological data depicting AH situation and its etiologies is missing in the center of Tunisia. To our knowledge, the impact of viral or non-viral etiology on the epidemiological, clinical and out-comes of AH has never been studied, so far, in a Tunisian population. Thus, we set ourselves the following goals: study the etiological spectrum of AH in Tunisian population and to specify the impact of viral etiology on the epidemiological, clinical and prognosis characteristics of AH.

## Methods

**Study design:** we conducted a cross-sectional study within the hepato-gastroenterology department of Sahloul Hospital collecting all patients with AH from January 2010 to December 2017. Patients who presented with the following criteria were included in the study: acute symptoms such as jaundice, fever, malaise, headache, nausea, vomiting, anorexia, diarrhoea, and abdominal pain; a serum alanine aminotransferase (ALT) level that was at least ten times the upper limit of normal without any history of pre-existing liver disease [4]. Patients with history of chronic hepatitis or with incomplete data were not included.

**Outcomes definition:** for each patient, the following data were collected: (i) *anamnestic data:* sex, age, risk factors such as history of transfusion, alcohol or drugs intake, contact with infected persons, (ii) *physical findings:* fever, HE according to West-Haven classification, cutaneous eruption, jaundice, enlarged liver or spleen, (iii) and *laboratory tests:* ALT, aspartate amino transferase (AST), prothrombine time (PT), international normalized ratio (INR), bilirubin levels and serum protein electrophoresis (SPE). We have specified the different etiologies of AH, the adopted treatment and the clinical outcomes (complications, ALF, death). AH was considered severe if PT was <50% or if INR was above 1.5 in the absence of vitamin K deficiency or the intake of vitamin K antagonist [5]. ALF was defined by an INR above 1.5 and any degree of hepatic encephalopathy (HE) in a patient without preexisting liver disease and with an illness of less than 26 weeks duration [1]. Fulminant hepatic failure (FHF) has been used to describe patients who develop HE within 2 weeks of the onset of illness. Subfulminant hepatic failure (SFHF) has been used to describe patients who develop HE more than 2 weeks but less than 26 weeks of the onset of illness [5, 6].

**Etiological diagnosis:** etiological diagnosis was based on the detailed clinical information and laboratory data. Viral etiology was confirmed by positive testing of serological markers of Hepatitis A, B, C or E viral infections, including IgM antibodies against HAV, HBV, hepatitis D virus (HDV), hepatitis E virus (HEV), anti-HCV antibodies and serum HCV RNA. Acute hepatitis A infection was defined as positive anti-HAV IgM, and acute hepatitis B was defined by a positive IgM anti-HBc and circulating levels of HBsAg [4]. Acute HCV infection was identified by positive HCV ribonucleic acid (RNA) in serum with either negative or positive anti-HCV antibody. HBV/HDV coinfection was diagnosed by positive HDV-IgM antibodies in patient with acute hepatitis and acute HDV superinfection was defined by positive HDV-IgM antibodies in patients chronically infected with hepatitis B [4].

In case of negative screening, non hepatotropic viruses such as Epstein-Barr virus (EBV), cytomegalovirus (CMV), Herpes simplex virus (HSV), and varicella zoster virus (VZV) infections and autoantibodies were searched. These include anti-nuclear antibodies (ANA), antiliver-kidney microsome antibodies (LKM-1) and anti-smooth muscle antibodies (ASMA) [4]. The implication of drugs or toxic was based on the detailed medical history at admission [7-9]. Diagnosis of Wilson's disease was based on the serum copper, ceruloplasmin levels, the presence of the Kayser-Fleisher ring, and family history of Wilson´s disease. Diagnosis of Budd-Chiari syndrome was based on abdominal imaging [10]. If no etiology was found, a liver biopsy was performed.

**Statistical analysis:** data were analysed by using SPSS (version 21.0, IBM). Categorical data were expressed as frequencies and percentages, while numerical data were expressed as means and standard deviations. Patients were divided in two groups: (i) patients with viral AH and (ii) patients with non-viral AH and then compared using chi-square test for qualitative variables and student test for quantitative variables. A p-value was considered as statistically significant if lower than 0.05.

**Ethical consideration:** no ethical approval was needed to analyse the patients' data. Nevertheless, we guaranteed that information were kept confidential and not personally identifiable.

## Results

**General characteristics:** we studied 129 files of patients with AH during an 8-year study period. Thirteen patients were excluded: 8 patients had unusable records, and 5 records were not found. Thirteen patients were not included due to a chronic liver disease history. Thus, 103 cases were retained. Patients were divided into 46 men and 57 women. The sex ratio was 0.8. The average age was 30.15 ± 15.86 years, ranging from 15 to 80 years old. Sixty patients (58.3%) were in the 15-25 age group with a slight female predominance (55.3%). Jaundice was the chief complaint, present in 81 patients (78.6%) followed by abdominal pain and asthenia in 41 (39.8%) and 30 (29.1%) patients respectively. Ninety-four (91.3%) patients had a cholestatic form and 8 (7.8%) had an anicteric form.

**Etiological diagnosis and management:** the etiology was identified in 92 patients (89.3%). The viral etiology was the cause in 70 patients (76.1%). AH is due to HAV in 52 of patients (74.1%), HBV in 16 patients (22.6%) and HCV in one patient (1.4%). One case (1.4%) of CMV hepatitis was noted. No case of hepatitis E was found. Elsewhere, it was toxic hepatitis in 10 patients (10.9%) including 7 drug-related AH. Budd-Chiari syndrome (BCS) and autoimmune hepatitis (AIH) with acute onset were noted in 3 (3.3%) and 7 (7.6%) patients, respectively. One case of Reye's syndrome and one case of thyrotoxicosis were identified. In the end, 11 patients (10.7%) have an indeterminate AH. Specific treatment has been initiated in: patients with severe viral HBV-related AH and received antiviral therapy; patients with acute onset AIH: corticosteroid therapy at a dose of 1 mg/kg/day; patients with AH secondary to BCS were put on anti-coagulant treatment at a curative dose and patient with thyrotoxicosis, had radioactive-iodine therapy then synthetic antithyroid drugs after rapid preparation with beta-blockers and corticosteroid therapy.

**Patients´ outcome:** the average duration of follow-up was 209.5 days, or approximately 7 months, with extremes ranging from 15 days to 6 years. Thirty-six patients (34.9%) were hospitalized in the hepato-gastroenterology department of Sahloul Hospital. The average hospital stay was 8.86 ± 5.62 days, with extremes ranging from 3 to 26 days. Sixty-nine (66.9%) patients had a hospital stay of less than 10 days. Ten (9.7%) patients presented a severe AH. Eight patients (7.8%) had ALF: 6 cases of FHF and 2 cases of SFHF (Table 1). Recovery was observed in 84 patients (81.6%). Evolution to chronicity was noted in 13 patients (12.6%). Five patients (4.9%) died from severe AH: 2 cases of toxic AH (one drug-related AH and one alcoholic AH), 2 cases of BCS and one case of AIH. Table 2 summarizes the patients´ outcome.

**Table 1:** presentations of acute hepatitis

	N	Mean age	Etiologies (N)
**Severe AH**	**10**	25.40±13.25 ans	**Reye’s syndrome (1)**
			**AIH (1)**
			**HVB (2)**
			**HVA (2)**
			**Drug-related AH (1)**
			**Toxic non drug-related AH (1)**
			**Indeterminate (2)**
**ALF**	**8**	39.38±20.45 ans	**Budd Chiari syndrome (3)**
			**AIH (1)**
			**HVB (1)**
			**HVA (1)**
			**Drug-related AH (1)**
			**AH due to Alcohol (1)**

**Table 2:** outcomes of patients with AH according to etiology

Etiology	Recovery	Chronicity	Death	Total
**viral acute hepatitis**	64 (91.4%)	6 (8.6%)	0 (0%)	70
HAV	52	0	0	52
HBV	11	5	0	16
HCV	0	1	0	1
CMV	1	0	0	1
**non viral acute hepatitis**	9 (40.9%)	8 (36.4%)	5 (22.7%)	22
Drug-related AH	6	0	1	7
Toxic non drug-related HA	1	1	1	3
AIH	0	6	1	7
Budd-Chiari Syndrome	0	1	2	3
Thyrotoxicosis	1	0	0	1
Reye’s Syndrome	1	0	0	1
**Indeterminate**	11 (100%)	0 (0%)	0 (0%)	11
**Total**	84 (81.6%)	13 (12.6%)	5 (4.9%)	103

**Analytic study:** we divided our patients into two groups: group 1 involved patients with viral AH including 70 patients (67.9%) while group 2 had patients with non-viral AH including 33 patients (32.1%). Table 3 and Table 4 summarize the comparison of qualitative and quantitative parameters respectively. Patients with viral AH were younger than those with non-viral AH with a statistically significant difference (25.36 years vs. 40.30 years; p = 10^-3^). Ascites was more common in group 2 vs group 1 (1 (1.4%) vs 7 (21.2%); p = 0.001). There was more hospitalization for non-viral AH (15 (21.4%) vs 21 (63.6%); p = 10^-3^) with a longer hospital stay (5.87 days ± 3.66 vs 11.00 days ± 5.87; p = 0.005). Patients with viral AH had a higher mean ALT level than those with non-viral AH (1601.41IU vs 1073.85 IU; p = 0.008). Liver damage was more severe in the non-viral AH group with lower PT (64.45% vs 81.38%; p = 0.001) and higher bilirubin level (197.81 umol/l vs 143.14 umol/l; p = 0.048). There were more severe forms (8.6% vs 18.2%; p = 0.013), more transition to chronicity (24.2% vs 8.6%; p = 10^-3^) and more deaths (15.2 %% vs 0%; p = 10^-3^) in the group of non-viral AH.

**Table 3:** comparison of qualitative parameters between the two groups

	Group 1 viral AH (N=70)	Group 2 No viral AH (N=33)	P
Age (Years)	25.36 ± 10.23	40.30 ± 20.49	**10-^3^**
Gender (Hommes/Femmes)	29/41	17/16	0.337
Urban environment (N)	37 (52.9%)	16 (48.5%)	0.679
Rural environment (N)	33 (47.1%)	17 (51.5%)	0.679
**Clinical signs**			
Jaundice (N)	66 (94.3%)	29 (87.9%)	0.595
Fever (N)	2 (2.6%)	3 (9%)	0.188
Right hypochondrium pain	12 (17.1%)	14 (42.4%)	**0.006**
Ascites (N)	1 (1.4%)	7 (21.2%)	**0.001**
Hépatomegaly (N)	5 (7.1%)	3 (9%)	0.709
**Clinical presentation**			
Cholestatic (N)	65 (92.9%)	30 (90.9%)	0.463
Common (N)	64 (91.4%)	21 (63.6%)	**0.01**
Severe (N)	4 (5.7%)	6 (18.2%)	0.071
ALF (N)	2 (2.9%)	6 (18.2%)	**0.013**
**Evolution**			
Recovery (N)	64 (91.4%)	20 (60.6%)	**10-^3^**
Chronicity (N)	6 (8.6%)	8 (24.2%)	**10-^3^**
Deaths (N)	0 (0%)	5 (15.2%)	**10-^3^**

**Table 4:** comparison of quantitative parameters between the two groups

	Group 1 viral AH (N=70)	Group2 No viral AH (N=33)	P
**Biological parameters**			
Hemoglobin (g/dl)	13.55±1.20	12.78±1.92	0.014
Platelets (10^3^ cell/mm^3^)	216.83±50.78	224.54±115.68	0.639
AST (IU/l)	1341.54±955.54	1061.64±897.89	0.161
ALT (IU/l)	1601.41±912.71	1073.85±964.02	**0.008**
AST/ALT >1 (N)	0 (0%)	18(54.5%)	**0.001**
PT (%)	81.38±20.53	64.45±28.13	**0.001**
Bilirubin level (µmol/l)	143.14±94.87	197.18±179.65	**0.048**
**Consultation delay (days)**	7.69±4.47	8.04±5.76	0.776
**Recorse to hospitalization**	15 (21.4%)	21 (63.6%)	**10-^3^**
**Hospital stay (days)**	5.87±3.66	11.00±5.87	**0.005**

## Discussion

This study is a large single centre experience of the etiological spectrum of AH and its outcomes. Several studies have focused on comparing the etiological, therapeutic and evolutionary aspects of ALF, however, few studies have focused on the impact of the viral etiology of AH on its clinical presentation and its outcome. The most important outcome of the present study is that, despite significant changes in socioeconomic parameters, the epidemic of acute viral hepatitis remains largely not decreased.

In our study, AH due to HAV affected 50.5% of patients with a female predominance. In fact, the HAV is the most common cause of viral AH and is responsible for 50 to 70% of cases of AH [11, 12]. Rich countries with good hygienic conditions have a very low endemicity but a higher proportion of adults likely to have viral AH with severe or even ALF. According to the last national survey carried out in 2016 in Tunisia, the epidemiological profile has changed: Tunisia is actually considered a country of low endemicity [13, 14]. HBV represented the second cause of AH with a frequency of 15.6% in our study, with a male predominance. About 12.5% had developed a severe form and 6.3% had developed ALF. However, no deaths had been reported. These results are consistent with those of a study carried out by Hellara *et al*. in a central region of Tunisia having included 105 patients, where the HAV was responsible for 51.4% of viral AH and the HBV was responsible of 38% of viral AH [3]. HCV is responsible for around 15% of viral AH [2, 15]. Most patients with AHC are asymptomatic (in 80% of cases) but a high proportion of patients progresses to chronicity, between 60 and 90%, depending on spontaneous viral elimination [12]. Severe AH is exceptional [2, 15].

With our study, a case of viral AH C was identified and had progressed to chronicity. In developing countries where genotypes 1 and 2 are endemic, HEV is responsible for symptomatic AH in young adults (15-30 years) [16-19]. Severe forms are seen in patients with underlying liver disease and in pregnant women where mortality can reach 25% in the third trimester of pregnancy [16-19]. In Tunisia, infection with the HEV has been little studied. Seroprevalence studies show that the prevalence of hepatitis E is much lower than that of hepatitis A [14]. The seroprevalence of hepatitis E among pregnant women in Sousse region was 12.1% [20]. In the study by Hellara *et al*. HEV was the cause of AH in 5.7% of cases [3]. In our study, no case of AH E was found.

Despite an exhaustive etiological assessment, in a significant proportion of patients (around 15 to 20%), no cause could be highlighted [16]. In our study, 11.7% of AH cases had an undetermined cause. The jaundice was the chief complaint, followed by abdominal pain and asthenia in 81 (78.6%), 41 (39.8%) and 30 (29.1%) patients respectively. These results are consistent with those of the literature which showed that jaundice and asthenia were the main reasons for consultation during AH [21, 22]. The presence of these symptoms is more frequent in the case of viral AH A (80% of adult patients) or B (30-50%) than in viral AH C (20%). According to Bernal *et al*. the initial manifestation of AH can range from nonspecific symptoms including malaise, anorexia, fatigue, nausea, vomiting and abdominal pain, to severe hypotension, sepsis, convulsion and HE [6]. Extrahepatic manifestations such as pleurisy, pericarditis, bone marrow aplasia or hemolytic anemia may be seen in rare cases [3, 23, 24]. In our study, none of our patients presented this type of clinical manifestation.

Our study also showed that, ALTs were also higher than ASTs in 82.5% of cases. However, the absolute values of transaminases have little correlation with clinical severity and the risk of liver failure [5, 16]. Nevertheless they can give an etiological orientation. Indeed, when cytolysis predominates over aspartate transaminases (ASTs), it is necessary to evoke toxic, hypoxic, herpetic AH and Budd-Chiari syndrom [5]. Similarly, the level of transaminases may suggest certain etiologies [2]. Indeed, a high level of transaminases around 1000 to 3000 IU evokes in the first place a viral AH or an AH due to paracetamol [25]. Although the increase caused by hepatitis C is often less important than that of hepatitis A or B [1, 2, 5, 11]. In our study, patients with viral AH had higher ALT level than the non-viral AH group (1601.41 IU vs 1073.85 IU; p = 0.008, CI = 95%) with an AST/ALT ratio > 1 in 54.5% of them (p = 0.00 1, CI = 95%).

The prognosis of AH is variable. The severity of AH can be related to the degree of liver functions impairment, the rapidity of progression of the disease (acute vs sub-acute course), the cause, the existence of chronic underlying liver disease and any associated comorbidities [1]. Until the years 1995-1996, viruses were the main causes of ALF (HAV> HBV> HEV> HCV) [1, 16]. Since then, drugs and in particular paracetamol have become the main cause in Europe and the United States [1, 10]. These results are in agreement with those of our study, where we found more ALF in the group of non-viral AH than in the group of viral AH with a statistically significant difference (p=10^-3^). It is currently recognized that some etiologies of ALF have a good prognosis. Among these etiologies, we find paracetamol (30% spontaneous survival), hepatitis A (43%) and shock liver [1]. The rate of spontaneous ALF resolution in acute hepatitis A is about 70%, the remaining 30% will require a liver transplant or [26]. During our study, patients with hepatitis A had a good evolution, even for those with a severe form. However, drug-related ALF not due to paracetamol (idiosyncratic drug reaction), autoimmune ALF and those due to HBV have a 1-year survival of less than 20% without liver transplantation and are therefore considered to have a poor prognosis. Non-advanced age, body mass index within normal limits, stage I-II HE were considered to be good prognostic factors during ALF [27]. In addition, other studies have shown that the concomitant use of alcohol and paracetamol can also contribute to progression to a severe form [28]. However, none of these factors was present in our patients.

**Strengths and limitations:** our study is the first retrospective study to our knowledge in Tunisia, raising the etiological, prognostic and therapeutic problems of AH. The results found in our study concerning the distribution of the etiologies of AH as well as their evolutionary aspects are consistent with the literature with a tendency to approach European epidemiological data in recent years. However, our study has some limits: in fact, we could not calculate an annual incidence since we have no idea about the number of cases diagnosed and not referred for exploration and on the other hand, the asymptomatic forms of AH are frequent. We agree that only a national epidemiologic study can provide a definitive answer regarding the overall prevalence and incidence of AH and dynamic trends over time. Therefore, these results should be later confirmed in multicenter studies with a larger population study and greater statistical power.

## Conclusion

Our work is the first Tunisian study to look at the impact of viral etiology on the epidemiological, clinical, biological but evolutionary characteristics of acute hepatitis. It demonstrated that the viral etiology is still the first cause of AH especially due to HAV and HBV and that it affects more likely young people. According to our study, non-viral AH (drug related AH, BCS) are associated with more severe forms, more transition to chronicity and more deaths compared to patients with viral AH. However, the etiology of AH remains unknown in a non-negligible proportion of patients. Our study lead us to insist on few points to improve the prognosis of AH such as vaccination against HBV and HAV, urgent need for efforts to improve infection control measures in the medical system and promoting liver transplantation.

### What is known about this topic

- Acute hepatitis is a major public health problem;
- The most commonly reported etiologies of AH are viral and drug-related ones.

### What this study adds

- The viral etiology is still the first cause of acute hepatitis;
- There were more severe forms and more transition to chronicity and more deaths in the non-viral AH group.

## References

[1] Bernal W, Wendon J (2013). Acute Liver Failure. N Engl J Med.

[2] Potel G, Batard E, Conte PL, Montassier E, Goffinet N, Berthier F (2013). Hépatites aiguës aux urgences, urgences.

[3] Hellara O, Melki W, Mastouri M, Ben Chaabène N, Loghmari H, Ben Mansour W Serodiagnosis of acute hepatitis in adults patients: results of a prospective study in a central region of Tunisia.

[4] OMS (2016). OMS | Considérations techniques et définitions de cas destinées à améliorer la surveillance de l´hépatite virale: rapport technique.

[5] Acute Liver Failure Guidelines EASL-The Home of Hepatology.

[6] Bernuau J, Rueff B, Benhamou JP (1986). Fulminant and subfulminant liver failure: definitions and causes. Semin Liver Dis.

[7] Navarro VJ, John R (2006). Drug-Related Hepatotoxicity. N Engl J Med.

[8] Bégaud B, Evreux JC, Jouglard J, Lagier G (1985). Imputation of the unexpected or toxic effects of drugs, Actualization of the method used in France. Therapie.

[9] Dangoumau J, Evreux JC, Jouglard J (1978). Mehtod for determination of undesirable effects of drugs. Therapie.

[10] Menon KVN, Shah V, Kamath PS (2004). The Budd-Chiari Syndrome. N Engl J Med.

[11] Sedhom D, D´Souza M, John E, Rustgi V (2018). Viral Hepatitis and Acute Liver Failure: Still a Problem. Clin Liver Dis.

[12] World Health Organization (WHO) Hepatitis.

[13] Société Tunisien de pathologie infectieuse Présentation des résultats de l’Enquête Nationale de Prévalence des Hépatites Virales A, B & C en Tunisie, 2015-2016.

[14] Safer L, Chaabane N, Melki W, Saffar H (2006). Épidémiologie des hépatites virales en Tunisie. Rev Epidemiol Sante Publique Rev Epidemiol Sante Publique.

[15] Thawley V (2017). Acute Liver Injury and Failure. Vet Clin North Am Small Anim Pract.

[16] Asrani SK, Devarbhavi H, Eaton J, Kamath PS (2019). Burden of liver diseases in the world. J Hepatol.

[17] Kamar N, Dalton HR, Abravanel F, Izopet J (2014). Hepatitis E virus infection. Clin Microbiol Rev.

[18] Dalton HR, Bendall R, Ijaz S, Banks M (2008). Hepatitis E: an emerging infection in developed countries. Lancet Infect Dis.

[19] Marano G, Vaglio S, Pupella S, Facco G, Bianchi M, Calizzani G (2015). Hepatitis E: an old infection with new implications. Blood Transfus.

[20] (2010). Rapport annuel d'activités de la sous-direction de santé de base de Sousse 2010-2017. Sous direction de soins de santé de base Sousse.

[21] Lok ASF, McMahon BJ (2007). Chronic hepatitis B. Hepatology.

[22] Yu YD, Park GC, Park PJ, Choi YI, Hwang S, Song GW (2013). Cytomegalovirus infection-associated fulminant hepatitis in an immunocompetent adult requiring emergency living-donor liver transplantation: report of a case. Surg Today.

[23] Bernal W, Hyyrylainen A, Gera A, Audimoolam VK, McPhail MJW, Auzinger G (2013). Lessons from look-back in acute liver failure: A single centre experience of 3300 patients. J Hepatol.

[24] Schiødt FV, Lee WM (2003). Fulminant liver disease. Clin Liver Dis.

[25] Yoon E, Babar A, Choudhary M, Kutner M, Pyrsopoulos N (2016). Acetaminophen-Induced Hepatotoxicity: a Comprehensive Update. J Clin Transl Hepatol.

[26] Taylor RM, Davern T, Munoz S, Han S-H, McGuire B, Larson AM (2006). Fulminant hepatitis A virus infection in the United States: Incidence, prognosis, and outcomes. Hepatology.

[27] Reuben A, Koch DG, Lee WM, Acute Liver Failure Study Group (2010). Drug-induced acute liver failure: results of a U.S. multicenter, prospective study. Hepatol Baltim Md. Hepatology.

[28] Lee WM, Squires RH, Nyberg SL, Doo E, Hoofnagle JH (2008). Acute liver failure: summary of a workshop. Hepatology.

